# An international analysis of the price and affordability of beer

**DOI:** 10.1371/journal.pone.0208831

**Published:** 2018-12-17

**Authors:** Evan Blecher, Alex Liber, Corné Van Walbeek, Laura Rossouw

**Affiliations:** 1 Health Policy Center, Institute for Health Research and Policy, University of Illinois at Chicago, Chicago, Illinois, United States of America; 2 School of Economics, University of Cape Town, Cape Town, South Africa; 3 Economic and Health Policy Research, American Cancer Society, Atlanta, Georgia, United States of America; 4 Health Management and Policy Department, School of Public Health, University of Michigan-Ann Arbor, Ann Arbor, Michigan, United States of America; 5 Economics of Tobacco Control Project, Southern African Labour and Development Research Unit, University of Cape Town, Cape Town, South Africa; Universidade da Coruna, SPAIN

## Abstract

**Aims:**

To apply methods for measuring the affordability of beer in a large cross section of countries, and to investigate trends in affordability of beer over time.

**Methods:**

We use the Relative Income Price (RIP), which uses per capita GDP, to measure the affordability of beer in up to 92 countries from 1990 to 2016 (69 countries were included in 1990, however the survey has since grown to include 92 countries). In addition to affordability, we also investigate trends in the price of beer.

**Results:**

While beer is, on average, similarly priced in high-income (HICs) and low- and middle-income countries (LMICs), it is significantly more affordable in HICs. There is significant variation in both price and affordability in HICs and in LMICs. Beer has become cheaper in real terms in 49% (18/37) of HICs and 43% (20/46) of LMICs. Beer became more affordable in most HICs (RIP: 30/37 or 81%) and LMICs (RIP: 42/44 or 95%)

**Conclusions:**

The increased affordability over time of beer in most countries raises concerns about public health. Governments need to increase taxes on beer so that it becomes less affordable over time, in an effort to improve public health.

## Introduction

The rapid rise in non-communicable diseases (NCDs), especially in low and middle-income countries (LMICs), is proving to be a global crisis, with increasing health expenditures, premature deaths and preventable morbidity. Focusing only on mortality, approximately 60% of annual deaths globally are attributable to NCDs [1]. One of the major modifiable risk-factors for various non-communicable diseases is the excessive use of alcohol. Epidemiological evidence has causally linked alcohol use to the prevalence of various non-communicable diseases, including cancers, poor cardiovascular outcomes, liver disease and pancreatitis [2]. Excessive alcohol use has also been associated with communicable diseases, such an increased risk of tuberculosis and incident HIV infection [3–5]. For tuberculosis, these operate through both pathological and social pathways. The substantial health, social and economic costs associated with excessive alcohol use have led the WHO to develop their Global Strategy to reduce the harmful use of alcohol in 2010 [6], which has resulted in an increasing number of countries developing national policies that address excessive consumption [7].

Understanding the economic factors which drive alcohol demand is crucial to curb growing non-communicable and communicable disease rates. Numerous studies over the past decades have shown that the demand for alcohol is heavily influenced by changes in pricing [8]. By increasing the excise tax, policy makers are able to increase the retail price of alcohol, thereby making alcohol less affordable. Results from a meta-analysis show the price elasticity of demand for beer, wine and spirits to be -0.46, -0.69 and -0.80, respectively [8]. Whereas increases in price temper demand, an increase in income tends to increase the demand for alcohol. In another meta-analysis, Gallet [9] finds that the income elasticity of alcohol is positive (magnitude 0.20).

The literature has focused more on the role of price as an economic contributor to the demand for alcohol rather than income, given that policy can only be designed to affect price and not income. The unprecedented economic growth rates being experienced by countries in LMICs has resulted in an increase income and people’s purchasing power. Consequently, alcohol has become more affordable.

This paper focuses on the *affordability* of beer. While studies that estimate the demand for alcohol will typically include both price and income as independent variables in a regression equation and consumption as the dependent variable, the estimated coefficients are interpreted separately. Affordability considers price and income simultaneously. Although there are various methodological definitions of affordability, the concept essentially refers to the quantity of resources required to buy a unit of the product under investigation (in terms of time, money or other products). In this article, we use the literature on the affordability of cigarettes as a benchmark for considering the trends in the affordability of beer due to the paucity of peer-reviewed literature on alcohol or specifically beer affordability.

## Literature review

The first studies compared changes in the price of cigarettes to changes in the price of a Big Mac hamburger, because the Big Mac is an internationally standardized product [10, 11]. These were tongue-in-cheek studies, but they spawned a more serious literature in their wake.

A second approach adopted by Guindon et al. [12] used an explicit measure of income by considering the time worked to purchase a pack of cigarettes (the “minutes of labour” approach). The authors calculated the average number of working minutes required to purchase a pack of cigarettes using the weighted average hourly earnings of twelve occupations monitored by the Union Bank of Switzerland’s (UBS) survey of earnings.

Blecher and Van Walbeek [13, 14, 15] defined affordability in terms of per capita GDP (*relative income price* or RIP). They defined the RIP as the percentage of per capita GDP required buy a given quantity of cigarettes per year. A similar approach was adopted by Blecher and colleagues [16] in measuring the affordability of sugar-sweetened beverages (SSB). Consumption of SSBs have increased globally in past decades [17], posing a public health concern. Using the RIP methodology, the authors found that SSBs became more affordable over time.

For high income countries (HICs) the correlation between MoL and RIP is high but for LMICs it differs significantly [14]. Both measures of affordability take an aggregated approach, which can be problematic in countries where there are large dispersions in income or high levels of unemployment. As a result, there is a move towards more individualised measures of affordability. Nargis and co-authors [18] monitor the affordability of tobacco in Bangladesh by income groups using “International Tobacco Control Policy Evaluation Project” data. However, this approach would require household or individual level data sets.

Kan [19] added to the literature by focussing not on the average (or median) income of the hourly earnings as Guindon et al. had done, but by focussing on the 25^th^ percentile and thereby emphasising the affordability of the product among the poor.

The affordability of a consumer product should be considered relative to the affordability of another products. For instance, one within the same country at a point in time. The affordability of a product cannot be considered in absolute terms, so one cannot claim that a consumer product is affordable or not affordable. The relative affordability of a product will also be influenced by the choice of income measure given that some measures of income are more representative than others. Narrower measures of income, such as the salary provided by a single occupation, may prove problematic in making broad and generalizable statements about the affordability of a product. The measure runs the risk of saying more about the salary associated with the occupation than the affordability measure itself.

These studies have generally found that cigarettes are more affordable in HICs than LMICs, but that they have become less affordable in HICs and more affordable in LMICs over time. In LMICs with stronger tobacco control policies cigarettes have generally become less affordable.

A smaller literature investigating the affordability of alcohol products currently exists. Kan and Lau [20] investigate the affordability of various alcohol products for 65 cities in 2009 using the same methodology applied for cigarettes [19]. Although the authors find that alcohol is highly affordable in many cities (88%), they do not consider changes in alcohol affordability over time.

Nelson [21] investigates the affordability of alcohol in OECD countries and finds that alcohol has become more affordable between 1975 and 2008. Nelson determines that alcohol become more affordable due to rising incomes rather than falling prices. A report by RAND Europe also considers country level affordability in the European Union [22] and additional country level research has been conducted in the United States [23]. For the US, Kerr finds that alcohol became more affordable since the 1960s, driven largely by a drop in the real price of alcohol. Similarly, Rabinovich and co-authors find that the real price of alcohol declined in the EU [22].

Multinational beer producer SAB Miller has also recently published estimates of beer affordability in popular publications [24, 25]. Its measure, the average hours worked to purchase 500ml of beer, is analogous to Guindon et al.’s’ measure. The SAB Miller estimates show that beer is more affordable in HICs than in LMICs, ranging from 0.15 hours worked in the Netherlands to 7.7 hours in Mozambique, for 500 ml of beer.

## Methodology

### Data

Measuring affordability requires data on prices and incomes. Price data are drawn from the “Worldwide Cost of Living Survey” of the *Economist Intelligence Unit* (EIU), which is conducted every six months (every June and December) in order to assess the prices of goods and services in the world’s major cities. Prices used in this study were collected biannually and averaged, covering each year for the period 1990 to 2016. The EIU survey contains the largest number of countries in which the prices of cigarettes and alcohol are consistently collected annually over a significant period of time, which has made it a popular source of price data in the affordability literature.

For most countries, a single city is monitored. In countries where multiple cities are monitored, an unweighted average price is calculated. In 1990 the survey included 103 cities in 69 countries. Over the period of investigation, the EIU expanded the coverage of their survey to include additional cities in countries that were already represented in the survey in 1990. By 2013 the scope of the survey had expanded to 140 cities in 92 countries. The study excluded cities that were added to the EIU’s sample if that country was already monitored, since they could introduce an artificial structural break in the price series if the cost of living in the newly included city differs significantly from those in the original survey. In some countries alcohol products are not legally sold or not easily available because of religious objections. Prices are not collected in these countries. City-level price data is used as a proxy for national-level price data. This assumption is non-problematic if we assume that the variation of price between sampled cities and the rest of the country remains relatively stable over time. National level analysis is necessary given that fiscal policy on taxing policy is, across countries, largely set at a national level.

Two beer products are surveyed, a local beer brand (in units of 1 litre) and a premium or top-quality brand of beer (in units of 330ml). The survey considers the prices at two types of outlet: high volume supermarket, and mid-price retail outlet Consistent with the cigarette and sugar-sweetened beverages affordability literature [13, 14, 15, 16], the cheapest of these four observations are used for affordability calculations. Furthermore, this is particularly important in the beer market since there is significant public health concern with the availability of cheap beer (i.e. at the bottom of the distribution) rather than the typical beer.

The EIU collects price data in local currency. To compare alcohol *prices* between countries, all prices were converted to US dollars using market exchange rates on the day of the survey from the EIU. However, affordability measures can be calculated using the local currency, since both price and income data are reported in local prices. The EIU data is a suitable data set for this analysis. It is advantageous in that collection is standardized across countries and years, and it includes a wide range of countries (including LMICs) over a long period of time. One drawback of the data set is that it is limited to two brand prices, and two types of retail outlets.

Prices are tangible and thus easy for people to understand, but incomes are more difficult since one can define incomes broadly (e.g. per capita GDP) or narrowly (e.g. net income). For example: “A Johannesburg teacher’s net hourly earnings in 2016 were $6.92” is typically better understood than “Per capita GDP in South Africa in 2016 was $5,273”.

Following from the cigarette affordability literature of Blecher and Van Walbeek [13, 14] a broad measure of affordability is employed. Per capita GDP has the advantage of being calculated using a consistent methodology and is generally regarded as a good indicator of average living standards. It has the disadvantage that it does not take differences in the distribution of income into account. GDP data were drawn from the World Bank [26].

As a robustness check, a secondary measure of affordability is calculated, namely the median income in many occupations. The second income measure is the UBS survey of earnings (also used by Guindon et al. [12], Blecher and van Walbeek [14] and Kan [19]). The survey calculates gross and net hourly earnings for several occupations in key commercial cities around the world every three years. We used six surveys (1997, 2000, 2003, 2006, 2009 and 2012) to construct a discrete time series of median earnings. The surveys were based on twelve occupations in 1997 and 2000, thirteen in 2003, fourteen in 2006 and 2009 and fifteen in 2012. 55 cities in 48 countries were included in the 1997 survey, growing to 72 cities in 59 countries by 2012.

The data on median income is only available for the period 1997 to 2012, and only include data on 35 HICs and 20 LMICs. Due to this limited data availability, the analysis is reported in the appendix and only referred to in the paper. The analysis is included as the method may allow greater accuracy in countries with large income dispersions where the GDP per capita is driven up by a smaller group of high income earners. However, GDP per capita is a better reflection of the economic status of countries with significant social benefits, common in various middle-income countries. Social benefits are included in GDP per capita measures but not in wage estimations. Furthermore, using wages as a measure of income are not necessarily representative of countries with high levels of unemployment. Blecher and Van Walbeek’s analysis of cigarettes [12] showed that there was greater correlation between the methods in HICs than in LMICs, and the choice of methodology is therefore more important for LMICs than for HICs. The UBS survey is not necessarily representative of occupations in LMICs, since formal employment in these countries (and the proportion of people in the occupations monitored by the UBS) is much lower than in HICs.

### Methods

The RIP is a broad measure of affordability developed by Blecher and Van Walbeek [13] for measuring the affordability of cigarettes. This measure has been oft applied in the tobacco control literature [27, 28, 29, 30]. The RIP calculates the percentage of per capita GDP required to purchase the 100 cheapest packs of cigarettes. A high RIP indicates *less* affordability, and vice versa. The RIP for beer was calculated as the ratio of nominal price to nominal income for each year in the period 1990 to 2016, for as many countries as the data allowed. It is defined as the percentage of per capita GDP required to buy 100 cans of the cheapest beer. Both prices and income are exogenous in this calculation.

The second measure employing the UBS data is the *minutes of labour* (MoL) method, developed to measure the affordability of cigarettes by the World Health Organisation and popularised by Guindon et al. [12]. The method has also been applied in several peer-reviewed publications [29, 30]. It is defined as the MoL required to purchase the cheapest pack of cigarettes (as surveyed by the EIU), based on net earnings. While there are a number of variations of this methodology (see Guindon et al. [12]) we use the median net earnings for calculating the MoL affordability measure as it is less susceptible to outlier (compared to the mean).The World Bank’s classification in July 2016 was used to divide the countries into two income categories: HICs (40 for GDP and 35 for UBS) and LMICs (47 for GDP and 20 for UBS).

## Results

### Differences in beer prices in 2016

Since price and tax data are often easily accessible, these are typically the focus of cross-sectional studies. However, prices by themselves are not necessarily a good indicator of affordability. Nevertheless, given that prices form a large component of the affordability measures, we consider beer prices in some detail here.

**Fig 1:**
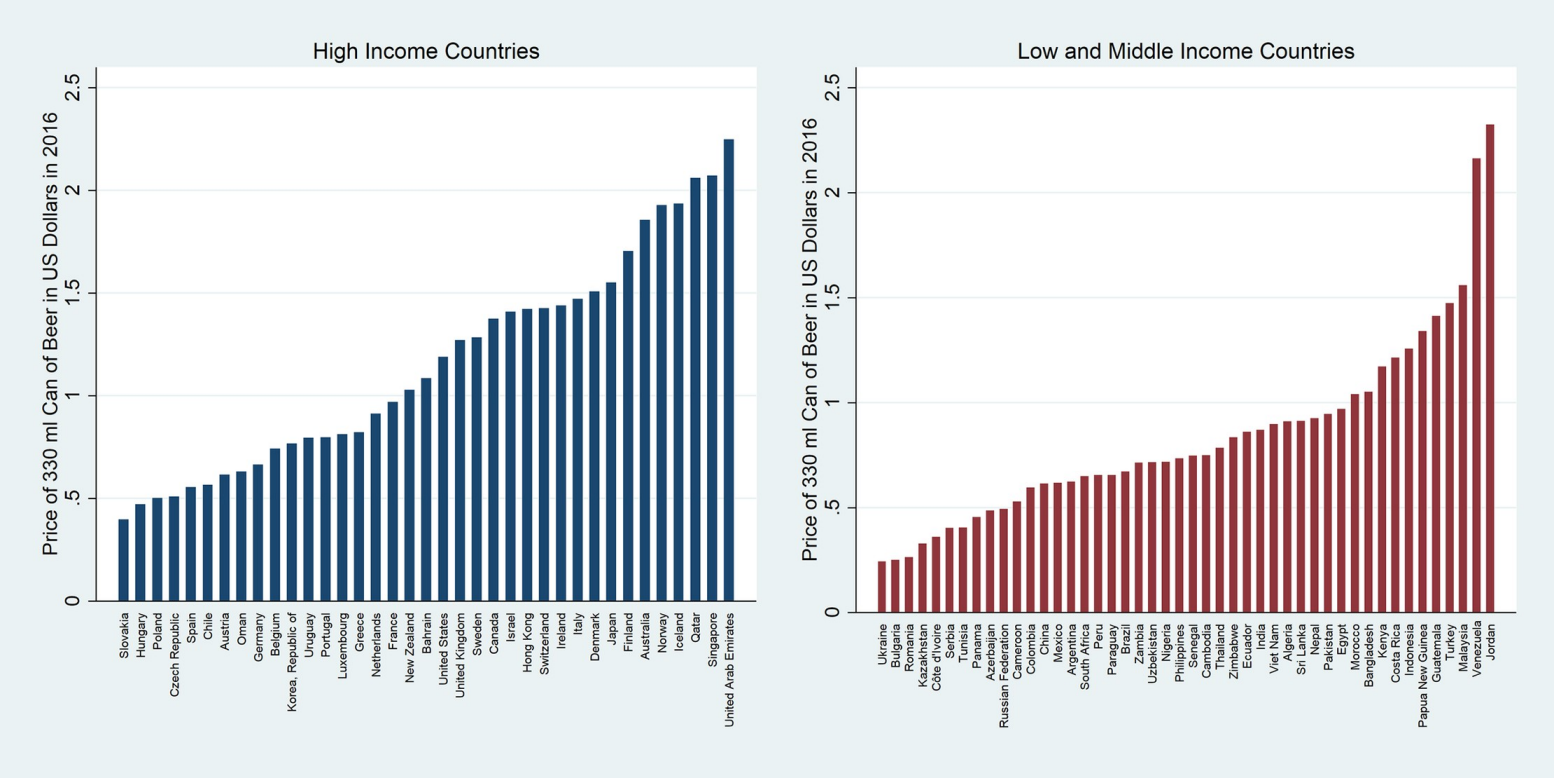
Nominal prices of beer in USD in 2016.

Fig 1 shows the lowest collected price of beer in nominal US Dollars for the most recent year, 2016. An alternate to nominal prices is purchasing power parity prices (PPP). PPP is a technique used to determine the relative values of different currencies such that using a PPP exchange rate would mean that the purchasing power of two countries to be the same. PPP might be considered a weak measure of affordability. However, it does not take the levels of income into account. The data are presented with countries ranked according to their development status (HICs and LMICs) and then according to the price from cheapest to most expensive. The top panel of Table 1 summarizes the measures of central tendency and variation for HICs and LMICs, respectively.

**Table 1:** Summary statistics for beer prices in 2016.

	Median	Mean	Std. Dev.	CV
Market exchange rates				
HICs	$1.09	$1.16	$0.52	0.45
LMICs	$0.74	$0.84	$0.44	0.53
LMIC as multiple of HIC	0.7	0.7		

Beer is, on average and at the median, more expensive in HICs (median = $1.09; mean = $1.16) than in LMIC (median = $0.74; mean = $0.84). A second feature is the very large variability in prices among countries with a similar level of development. While the standard deviation in HICs and LMICs is very similar ($0.52 vs. $0.44, respectively), the coefficient of variation (CV) (i.e. the ratio of the standard deviation to the mean) is higher in LMICs (0.45 vs. 0.53). While the figure is certainly useful in some circumstances (e.g. tourists knowing that alcohol is very expensive in some countries), one cannot infer anything about the affordability of beer, because it does not incorporate the level of income.

Rather than using market exchange rates to convert local currency prices to internationally standardised prices, one could use PPP conversion factors. This approach takes account of the fact that the costs of living vary between countries, but it still does not consider the impact of differences in the level of income between countries. As such, it is still a price measure rather than an affordability measure.

### Difference in affordability in 2016

**Fig 2:**
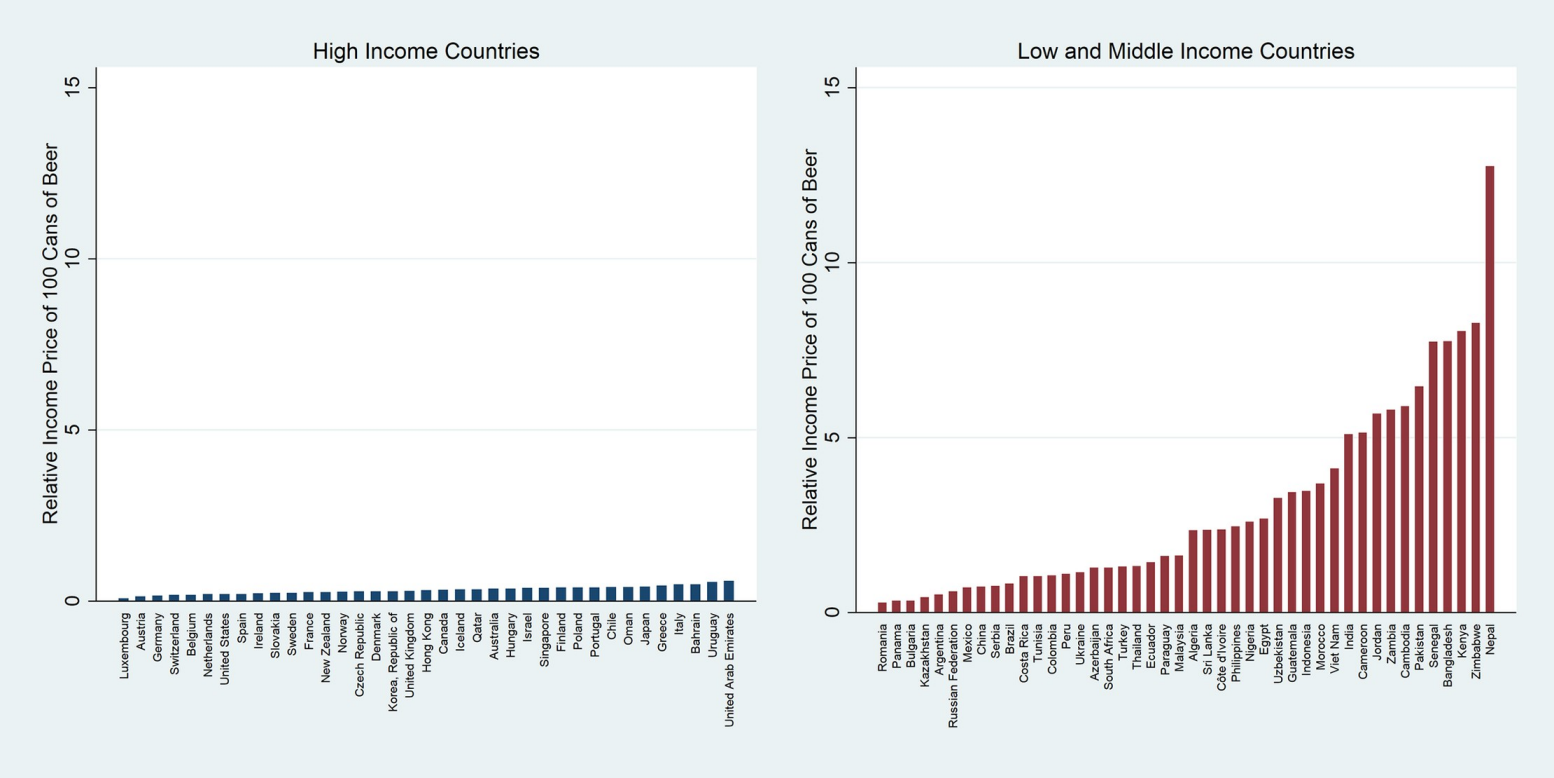
Relative income price (RIP) of beer in 2016 (same axis).

**Fig 3:**
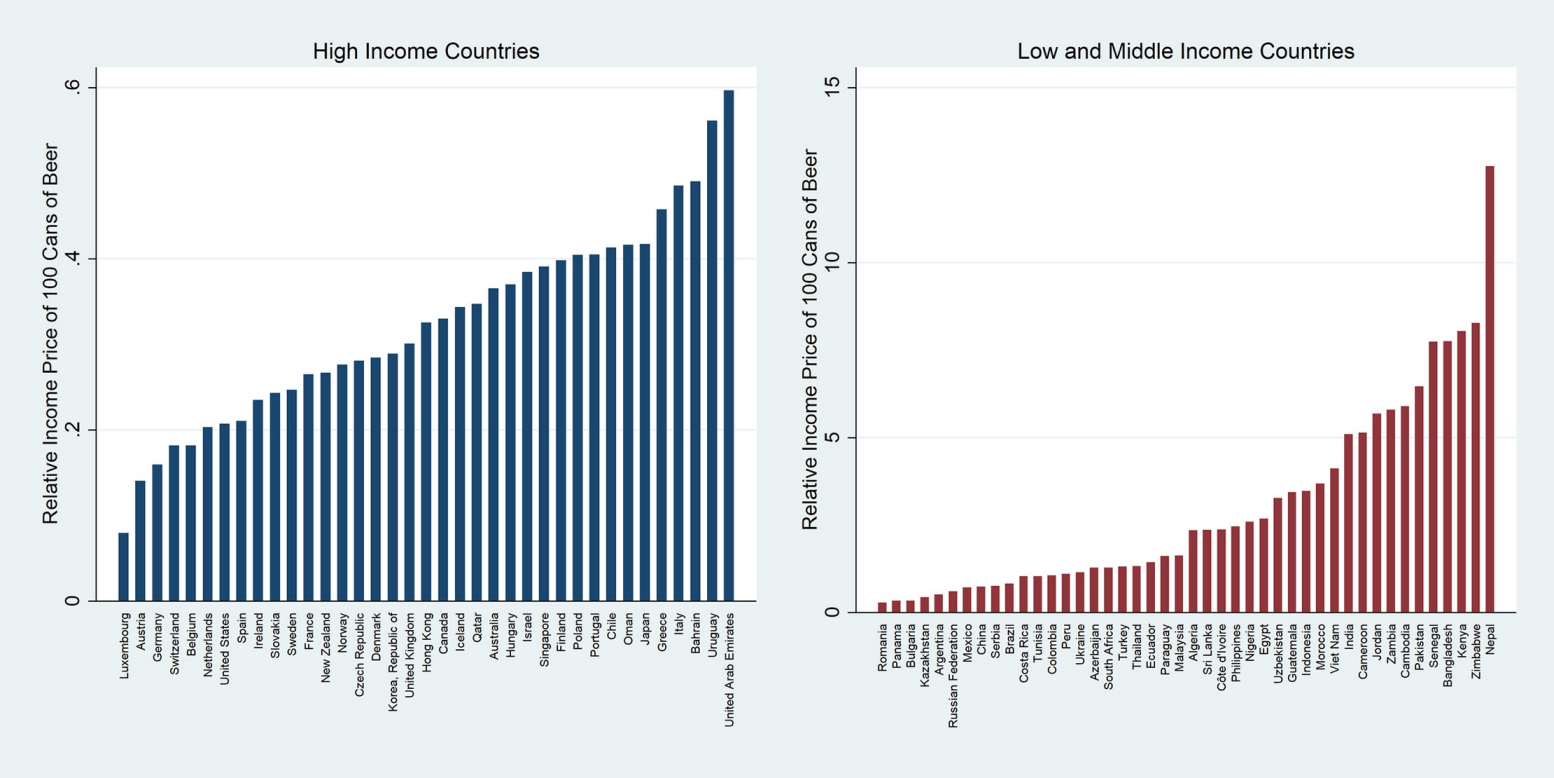
Relative income price (RIP) of beer in 2016 (dual axis).

The lower the relative income price (RIP), the more affordable beer is, and the higher the RIP, the less affordable it is. Similarly, the lower the minutes of labour (MoL), the less work time is required to purchase a given quantity of beer, and hence the more affordable it is. The RIPs in the most recent years are shown in Figs 2 and 3. Two figures are shown, the first with vertical axes similar in size, and the second with two different vertical axes, one for HICs and another for LMICs. The range of RIPs for LMICs is significantly higher than for HICs, and by using two different vertical axes, the reader to appreciate the range in the RIP across countries. Table 2 summarizes the measures of central tendency and variation of the affordability measure for HICs and LMICs.

**Table 2:** Summary statistics for beer affordability in 2016.

	Median	Mean	Std. Dev.	CV
RIP				
HICs	0.33%	0.32%	0.12%	0.36
LMICs	1.99%	3.01%	2.80%	0.93
LMIC as multiple of HIC	6.12	9.3		

When considering the RIP, beer is significantly more affordable in HICs (median = 0.33%; mean = 0.32%) than in LMICs (median = 1.99%; mean = 3.01%). The scale of the difference in affordability is very large; beer is, on average, approximately 9 times more affordable in HICs than LMICs. This is even though beer is more expensive in absolute terms in HICs. The explanation lies in the fact that per capita GDP in HICs exceeds per capita GDP (in common currency) in LMICs by a much greater multiple (8 times in 2016 for example).

The MoL affordability measure for beer is shown in S1 Fig. Table in S1 Table summarizes the measures of central tendency and variation of the MoL affordability measure for HICs and LMICs.

The relatively few LMICs which are included in the UBS dataset prevent us from making as strong conclusions about the MoL method as we did for the RIP. Again, beer is significantly more affordable in HICs (median MoL = 5.7; mean MoL = 5.8) than in LMICs (median MoL = 12.9; mean MoL = 18.1). For the MoL affordability measure the differences between HICs and LMICs are smaller than what they are for the RIP affordability measure. There is also a large variation in the MoL for beer, especially for the LMICs (CV = 0.79), but the variation is not as great as we saw for the RIP measure (CV = 0.93).

There is also a large range in the RIPs. The variation in RIPs is significantly higher than the variation in prices in these countries (CV for RIP for LMICs = 0.93, compared to CV for nominal prices for LMICs = 0.53). This increased variation is due to the significant variation in per capita GDP and shows that HICs are more homogeneous than LMICs. It also shows that it is more difficult to compare LMICs to each other.

### Trends in prices

**Fig 4:**
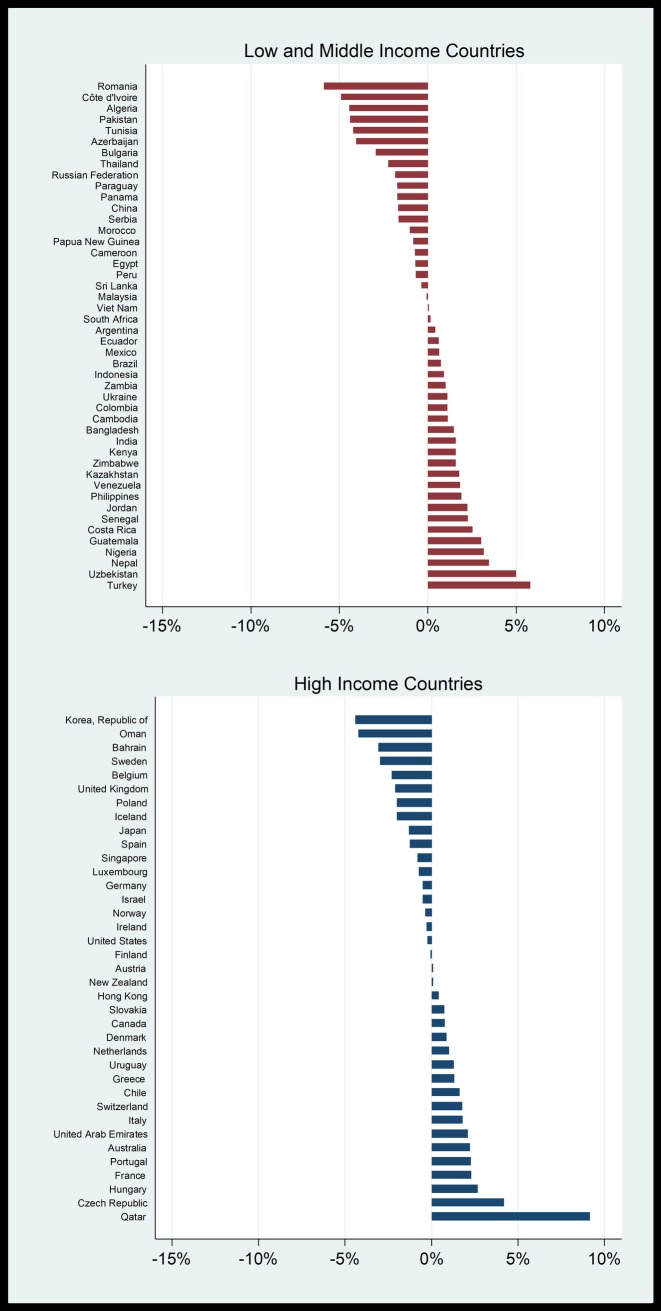
Average annual percentage change in real prices of beer, 1990–2016.

The previous discussion considered cross-sectional prices and affordability measures, while this section investigates the trends in prices since 1990.Some countries included in the analysis have shorter time periods. We consider real prices, rather than nominal price, as was the case previously. Nominal prices are converted to real (inflation-adjusted) prices using the consumer price index. The constant growth regression method is used to measure the average annual percentage change in real prices. This entails running the regression ln(P_t_) = α + ßt + u_t_, where ln(Pt) is the natural logarithm of the real price, t = 1, 2, …., n, and α and ß are the parameters. Positive price growth (ß>0) means that beer has become more expensive in real terms, while negative price growth (ß<0) means that beer has become less expensive in real terms. The trend analysis of real prices is done using local currencies. Fig 4 shows the growth in real beer prices for individual countries from 1990 to 2016, although the periods differ for some countries. Beer has become more expensive in 51% (19/37) of HICs, and in 57% (26/46) of LMICs. It has become cheaper in 49% (18/37) of HICs and 43% (20/46) of LMICs.

### Trends in affordability

The affordability of beer from 1990 to 2016 is estimated in this section. The constant growth regression method is used to measure the average annual percentage change in affordability. Positive growth means that beer has become less affordable, while negative growth means that beer has become more affordable.

**Fig 5:**
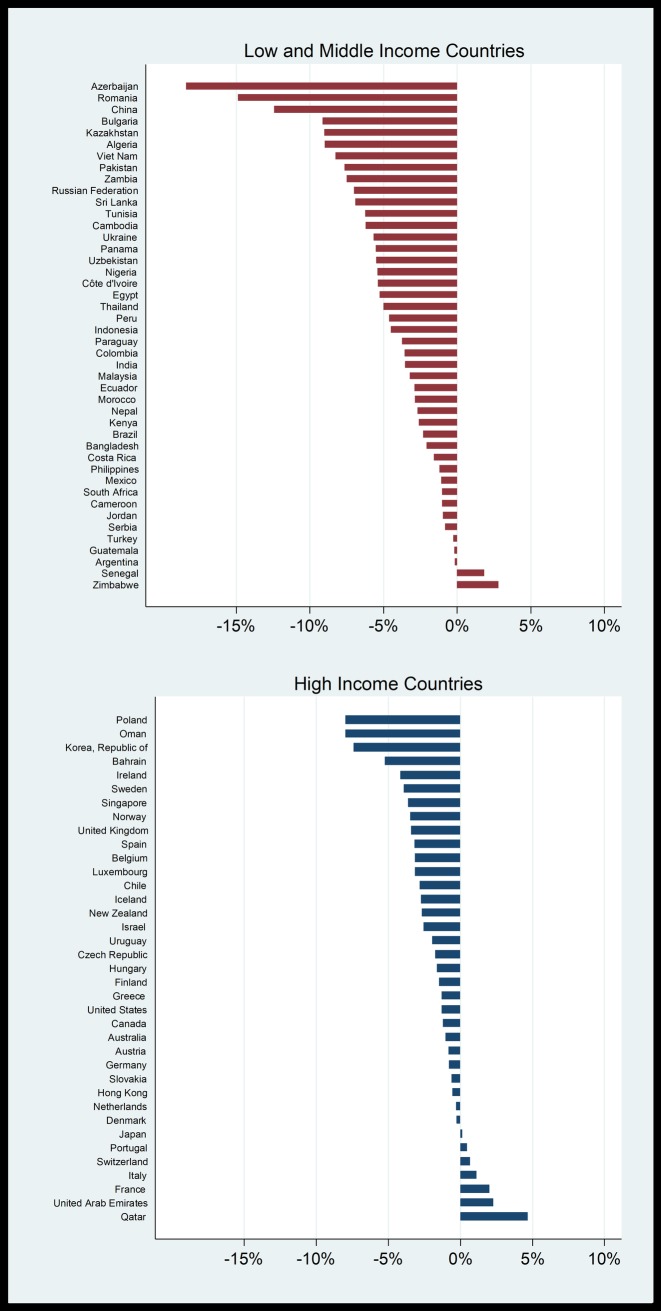
Average annual percentage change in affordability (RIP) of beer, 1990–2016.

Fig 5 shows the change in affordability in beer prices for individual countries from 1990 to 2016, using the RIP method.

Beer became more affordable in nearly all HICs and all LMICs. More specifically, beer became more affordable in 81% (30/37) of the HICs and in 95% (42/44) of the LMICs. This is likely to have been driven by the decrease in the real prices in many countries, combined with sizeable increases in average income levels in many countries (a more detailed decomposition is provided later in this section). In four HICs (Bahrain, Korea, Oman and Poland) and twenty LMICs (which include China, Russia, Pakistan and Nigeria as the four most populous countries) beer has become more affordable by an annual rate of 5% or more over the 1990–2016 period. S2 Fig shows the change in affordability in beer for individual countries from 1997 to 2012, using the MoL method. Using this method, we also find that beer has, on average, become more affordable in both HICs and LMICs, but that fewer countries experience this trend than with the RIP method. Beer became more affordable in 54% (19/35) of HICs and in 65% (13/20) of LMICs. This difference is ascribed to wages not rising as quickly as per capita GDP in many countries.

**Fig 6:**
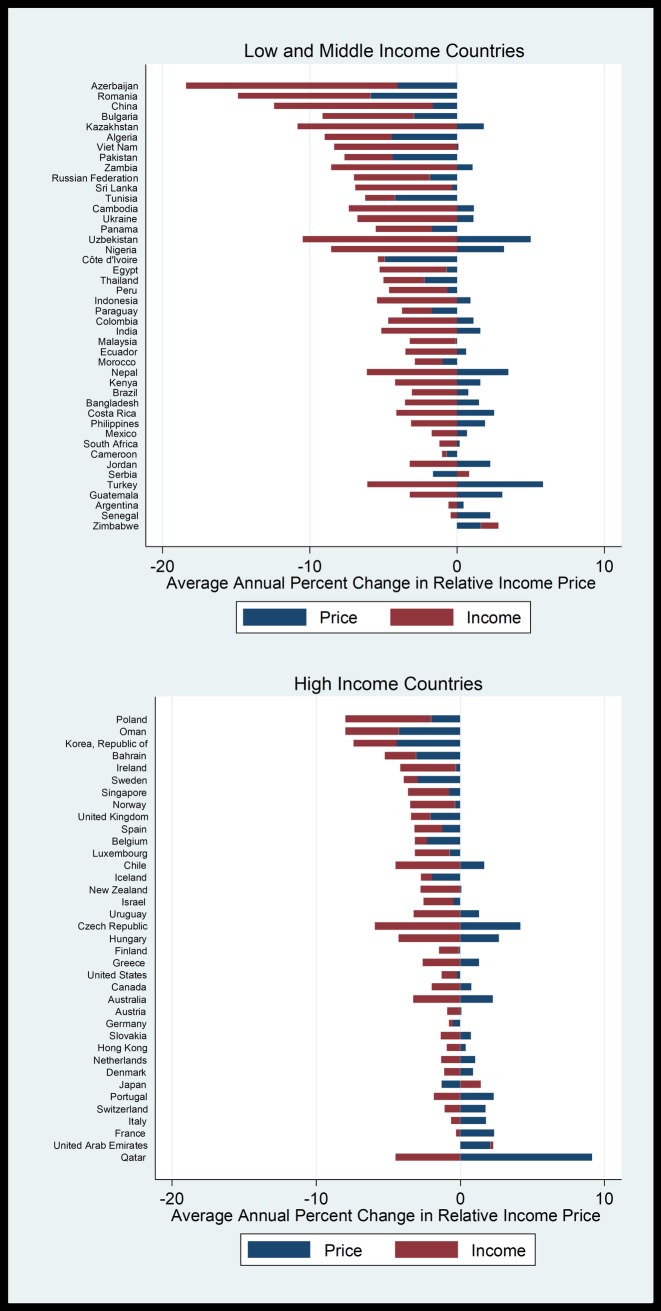
Decomposition of effects of income and price of beer in 37 high income countries and 44 low-income and middle-income countries from 1990 to 2016.

Finally, we decompose the changes in affordability into the changes in prices and changes in income. This allows us to pinpoint the driving factors of the decline in affordability. The decomposition is calculated by subtracting average annual percentage change of price (from section 4.3) from the average annual percentage change of RIP. The result can be attributed to the average annual percentage change of income. This is depicted in Fig 6.

Both price decreases and income increases contribute to the increase of beer affordability over time. In Azerbaijan, the LMIC where we see the largest shift in affordability, the effect was driven by both an increase in the country’s GDP per capita over time and a decrease in the real price of beer. In Nigeria, on the other hand, the real price of beer has increased over the study period. However, the relatively larger increase in the GDP per capita means that beer has become relatively more affordable.

While we observe price increases counteracting income increases in 19 of 37 HICs and 25 of 44 LMICs, the size of income increases in most of these countries were substantial enough to outweigh price increases and make beer more affordable. In a large proportion of countries, we observe a simultaneous decrease in price and increase in income (17 of 37 HICs and 18 of 44 LMICs), thereby increasing affordability. In these countries, we observe that the increase in affordability is more often driven by an increase in income (9 of the 17 HICs and 14 of the 18 LMICS) than a decrease in price, particularly in LMICs.

## Discussion and conclusion

The affordability of beer forms a crucial component of the demand for beer. This paper provides evidence of the increased affordability of beer in most countries over time, which raises concerns about public health. While both income and price influence affordability, increasing the excise tax on beer (resulting in an increase in beer prices) is the only tool that governments can use to decrease beer affordability and improve public health outcomes. However, monitoring both movements in price and income is necessary to gauge the size of price increase necessary to drive down demand.

While an increase in taxes that result in price increases are often a necessary condition to reduce the demand for beer, this can be crowded out by a relatively larger increase in income. Nelson [20] has shown that increases in affordability in OECD countries were driven by such increases in incomes. The crucial deduction from this is that the simultaneous analysis of both price and income allows a formal assessment of the affordability of beer across countries and across time. Furthermore, measuring affordability becomes more important with greater concern applied by the public health community with an increasing distribution of alcohol prices, particularly at the low end with cheap products now readily available in many countries, and their associated effect on public health (for example see [31]).

In this paper, we apply existing techniques for measuring the affordability of beer. Borrowing from the cigarette affordability literature we derived the RIP, which clearly shows the trend that beer has become more affordable in most countries in the world since 1990.

Beer producers consider affordability to be important, particularly in LMICs, where more rapid economic growth encourages growth in consumption. Although there is a positive gradient between socio-economic status (SES) and alcohol use, there is a higher incidence of the harmful consequence of excessive use among lower SES groups [32]. As alcohol becomes more affordable and more accessible, these groups become increasingly vulnerable. In two recent articles in the *Economist* and the *Sunday Times* (a South African publication), beer affordability took center stage, as SAB Miller, the world’s second-largest brewer by revenue, measured the affordability of beer in several countries [24, 25]. Using the minutes of labour approach (similar to Guindon et al. [12]), SAB Miller calculated that an average consumer in Africa works between 2 and 6 hours to purchase 500ml of beer, compared to between 9 and 17 minutes in European countries and the United States.

Our research shows that there is significant scope for governments to take action by raising excise taxes on beer, thereby ensuring that beer becomes less affordable over time, or even simply to stop the increase in the affordability of beer. Although the impact of a tax increase on the price of beer will depend on the market structure and size of the tax pass-through, country-level analysis and systematic reviews have consistently shown that increasing alcohol prices lead to a drop in alcohol consumption [33, 34]. Additionally, governments may consider other models to reduce the affordability of beer including the use of minimum prices which would narrow the range of prices (see [35]). While minimum prices are likely to be effective in narrowing the range of prices by increasing prices at the lower end, the rents of such a policy would be transferred to the supply chain. An excise tax system that relies more on specific taxes rather than ad valorem taxes is likely to achieve a similar outcome with the rents rather being transferred to the government.

The study does suffer from several limitations. Firstly, it is limited to beer, and no other alcohol products. While one is not able to make generalizable statements about the affordability of alcohol, beer nevertheless is the most consumed alcohol product in many, if not most countries and as such is an important indicator of trends in the affordably of alcohol. The homogeneity in the product makes it easier to compare across countries too. Secondly, while the length of time used in this study is significant it does not exclude the possibility that alcohol may have been becoming more or less affordable in the years preceding 1990, and thus may not correctly represent the longer-term trends in affordability. Thirdly, there is the issue of income distribution. Large income dispersions are not adequately captured in the average income measures used RIP calculations. The comparison of two countries with very different income distributions may prove problematic. Given the same price, beer is likely to be more affordable in a middle-income country with a relatively equal income distribution than in a country with a similar average level of income, but with high levels of poverty. Individualized affordability measures like those calculated by Nargis and co-authors [18] for tobacco in Bangladesh is an alternative, more comprehensive approach for this purpose (data permitting). Fourthly, factors, such as changes in currencies, hyperinflation, temporary spikes in prices, errors in collection, volatile exchange rates, etc., could always affect the accuracy of data. Reasonable measures have been taken to ensure that the data are correct. Finally, a major drawback of the current data set is that we are unable to perform the analysis for specific vulnerable sub-groups, such as the youth.

## Supporting information

S1 Table Summary statistics for beer MoL affordability in 2016. (PDF)

S1 Fig Minutes of Labour (MoL) to purchase beer in 2012. (PDF)

S2 Fig Average annual percentage change in affordability (MoL) of beer, 1997–2012. (PDF)

## References

[1] Bloom DE, Cafiero E, Jané-Llopis E, Abrahams-Gessel S, Bloom LR, Fathima S, et al The global economic burden of noncommunicable diseases. Program on the Global Demography of Aging; 2012 1.

[2] Parry CD, Patra J, Rehm J. Alcohol consumption and non‐communicable diseases: epidemiology and policy implications. Addiction. 2011 10 1;106(10):1718–24. 10.1111/j.1360-0443.2011.03605.x 21819471 10.1111/j.1360-0443.2011.03605.x PMC3174337

[3] Baliunas D, Rehm J, Irving H, Shuper P. Alcohol consumption and risk of incident human immunodeficiency virus infection: a meta-analysis. International journal of public health. 2010 6 1;55(3):159–66. 10.1007/s00038-009-0095-x 19949966 10.1007/s00038-009-0095-x

[4] Lönnroth K, Williams BG, Stadlin S, Jaramillo E, Dye C. Alcohol use as a risk factor for tuberculosis–a systematic review. BMC public health. 2008 12;8(1):289. 18702821 10.1186/1471-2458-8-289 PMC2533327

[5] Rehm J, Samokhvalov AV, Neuman MG, Room R, Parry C, Lönnroth K, et al The association between alcohol use, alcohol use disorders and tuberculosis (TB). A systematic review. BMC public health. 2009 12;9(1):450. 19961618 10.1186/1471-2458-9-450 PMC2796667

[6] World Health Organization, World Health Organization. Global strategy to reduce the harmful use of alcohol. 2010 Geneva: World Health Organization. 10.2471/BLT.19.241737 PMC7047030 32132758

[7] World Health Organization, World Health Organization. Management of Substance Abuse Unit Global status report on alcohol and health, 2014. World Health Organization; 2014.

[8] Wagenaar AC, Salois MJ, Komro KA. Effects of beverage alcohol price and tax levels on drinking: a meta‐analysis of 1003 estimates from 112 studies. Addiction. 2009 2 1;104(2):179–90. 10.1111/j.1360-0443.2008.02438.x 19149811 10.1111/j.1360-0443.2008.02438.x

[9] Gallet CA. The demand for alcohol: a meta‐analysis of elasticities. Australian Journal of Agricultural and Resource Economics. 2007 6 1;51(2):121–35.

[10] Lal A, Scollo M. Big Mac index of cigarette affordability. Tobacco Control. 2002 9 1;11(3):280–2. 10.1136/tc.11.3.280-b PMC1759037 12198286

[11] Scollo M. The Big Mac index of cigarette affordability. Tobacco Control. 1996 3;5(1):69. 10.1136/tc.5.1.69a PMC1759488 8795863

[12] Guindon GE, Tobin S, Yach D. Trends and affordability of cigarette prices: ample room for tax increases and related health gains. Tobacco control. 2002 3 1;11(1):35–43. 10.1136/tc.11.1.35 11891366 10.1136/tc.11.1.35 PMC1747639

[13] Blecher EH, Van Walbeek CP. Cigarette affordability trends: an update and some methodological comments. Tobacco Control. 2009 6 1;18(3):167–75. 10.1136/tc.2008.026682 19179369 10.1136/tc.2008.026682

[14] Blecher EH, Van Walbeek CP. An international analysis of cigarette affordability. Tobacco Control. 2004 12 1;13(4):339–46. 10.1136/tc.2003.006726 15564616 10.1136/tc.2003.006726 PMC1747952

[15] Blecher E, Ross H, Leon ME. Cigarette affordability in Europe. Tobacco Control. 2013 7 1;22(4):e6–e6. 10.1136/tobaccocontrol-2012-050575 23092885 10.1136/tobaccocontrol-2012-050575

[16] Blecher E, Liber AC, Drope JM, Nguyen B, Stoklosa M. Peer Reviewed: Global Trends in the Affordability of Sugar-Sweetened Beverages, 1990–2016. Preventing chronic disease. 2017;14. 10.5888/pcd14.160406 PMC5420443 28472607

[17] Duffey KJ, Popkin BM. Shifts in patterns and consumption of beverages between 1965 and 2002. Obesity. 2007 11 1;15(11):2739–47. 10.1038/oby.2007.326 18070765 10.1038/oby.2007.326

[18] Nargis N, Stoklosa M, Drope J. The Trend in Affordability of Tobacco Products in Bangladesh 2009–2015: Evidence from ITC Bangladesh Surveys. The International Tobacco Control Policy Evaluation Project. 2016.

[19] Kan MY. Investigating cigarette affordability in 60 cities using the cigarette price-daily income ratio. Tobacco control. 2007 12 1;16(6):429 10.1136/tc.2007.020487 18048622 10.1136/tc.2007.020487 PMC2807202

[20] Kan MY, Lau M. Comparing alcohol affordability in 65 cities worldwide. Drug and alcohol review. 2013 1 1;32(1):19–26. 10.1111/j.1465-3362.2012.00476.x 22672655 10.1111/j.1465-3362.2012.00476.x

[21] Nelson JP. Alcohol Affordability and Alcohol Demand: Cross‐Country Trends and Panel Data Estimates, 1975 to 2008. Alcoholism: Clinical and Experimental Research. 2014 4 1;38(4):1167–75. 10.1111/acer.12345 24717100

[22] Rabinovich L, Hunt P, Staetsky L, Goshev S, Nolte E, Pedersen JS, et al Further Study on the affordability of alcoholic beverages in the EU: A Focus on Excise Duty Pass-Through, On-and Off-Trade Sales, Price Promotions and Statutory Regulations. Rand health quarterly. 2012;2(2 10.7249/rhq-02-02-17 PMC4945281 28083258

[23] Kerr WC, Patterson D, Greenfield TK, Jones AS, McGeary KA, Terza JV, et al US alcohol affordability and real tax rates, 1950–2011. American journal of preventive medicine. 2013 5 1;44(5):459–64. 10.1016/j.amepre.2013.01.007 23597808 10.1016/j.amepre.2013.01.007 PMC3631317

[24] The beer frontier [document on the Internet]. The Economist. 2014 May 31 [cited 26 June 2018]. Available from https://www.economist.com/business/2014/05/31/the-beer-frontier

[25] Crotty A. Headwinds blow froth from the beer. Sunday Times South Africa [Internet]. 2014 5 25 [cited 26 June 2018]. Available from: https://www.pressreader.com/south-africa/sunday-times/20140525/282282433340697

[26] World Bank. World Development Indicators. 2016 [cited 26 June 2018]. Available from https://data.worldbank.org/products/wdi

[27] Guindon G, Hien N, Kinh H, McGirr E., Trung D, Lam N. Tobacco Taxation in Vietnam. Paris: International Union Against Tuberculosis and Lung Disease; 2010.

[28] Bandi P, Blecher E, Cokkinides V, Ross H, Jemal A. Cigarette affordability in the United States. nicotine & tobacco research. 2013 2 14;15(9):1484–91. 23410802 10.1093/ntr/nts348

[29] Mackay J, Eriksen M, Shafey O. The Tobacco Atlas American Cancer Society. Atlanta, Georgia, USA 2006.

[30] Eriksen M, Mackay J, Ross H. The tobacco atlas: American cancer society. Atlanta. 2012.

[31] Black H, Gill J, Chick J. The price of a drink: levels of consumption and price paid per unit of alcohol by Edinburgh's ill drinkers with a comparison to wider alcohol sales in Scotland. Addiction. 2011 4 1;106(4):729–36. 10.1111/j.1360-0443.2010.03225.x 21134019 10.1111/j.1360-0443.2010.03225.x PMC3085000

[32] Grittner U, Kuntsche S, Graham K, Bloomfield K. Social inequalities and gender differences in the experience of alcohol-related problems. Alcohol and alcoholism. 2012 4 27;47(5):597–605. 10.1093/alcalc/ags040 22542707 10.1093/alcalc/ags040 PMC3417684

[33] Jiang H, Livingston M. The dynamic effects of changes in prices and affordability on alcohol consumption: an impulse response analysis. Alcohol and Alcoholism. 2015 6 12;50(6):631–8. 10.1093/alcalc/agv064 26071563 10.1093/alcalc/agv064

[34] Sharma A, Sinha K, Vandenberg B. Pricing as a means of controlling alcohol consumption. British medical bulletin. 2017 9 1:1–0. 10.1093/bmb/ldx020 28910991

[35] Purshouse RC, Meier PS, Brennan A, Taylor KB, Rafia R. Estimated effect of alcohol pricing policies on health and health economic outcomes in England: an epidemiological model. The Lancet. 2010 4 17;375(9723):1355–64. 10.1016/S0140-6736(10)60058-X 20338629

