# Supplementary material for: An international analysis of the price and affordability of beer
Source: PLoS One. 2018 Dec 17;13(12):e0208831. doi: 10.1371/journal.pone.0208831 (PMC6296500; doi:10.1371/journal.pone.0208831)
Supplement: S1 Table — (PDF) [file pone.0208831.s001.pdf]

S1 Table. Summary statistics for beer MoL affordability in 2016

	Median	Mean	Std. Dev.	CV
**MoL**				
HICs	5.7	5.8	2.8	0.49
LMICs	12.9	18.1	14.3	0.79
LMIC as multiple of HIC	2.3	3.1		
