# Supplementary figures and images for: An international analysis of the price and affordability of beer
Source: PLoS One. 2018 Dec 17;13(12):e0208831. doi: 10.1371/journal.pone.0208831 (PMC6296500; doi:10.1371/journal.pone.0208831)

**S1 Fig. Minutes of Labour (MoL) to purchase beer in 2012**

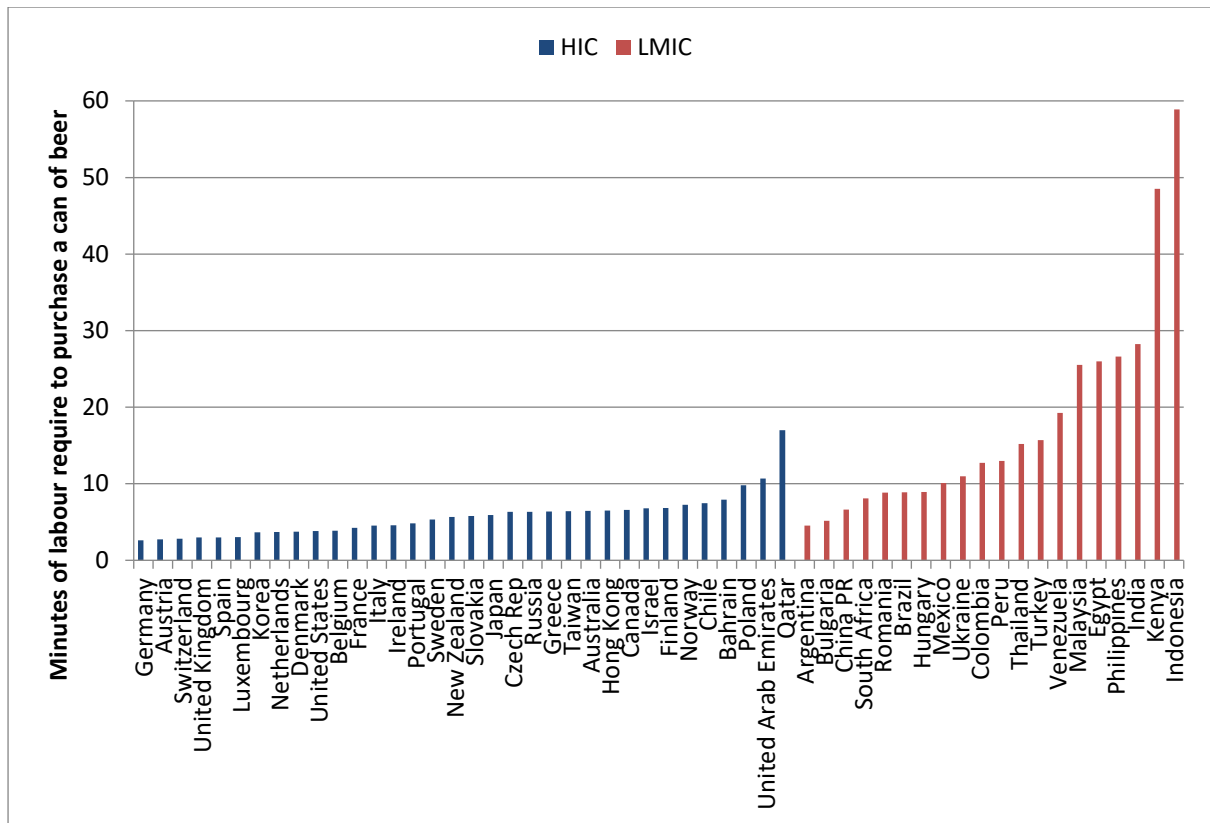

Supplement: S1 Fig — (PDF) [file pone.0208831.s002.pdf]

**S2 Fig. Average annual percentage change in affordability (MoL) of beer, 1997-2012**

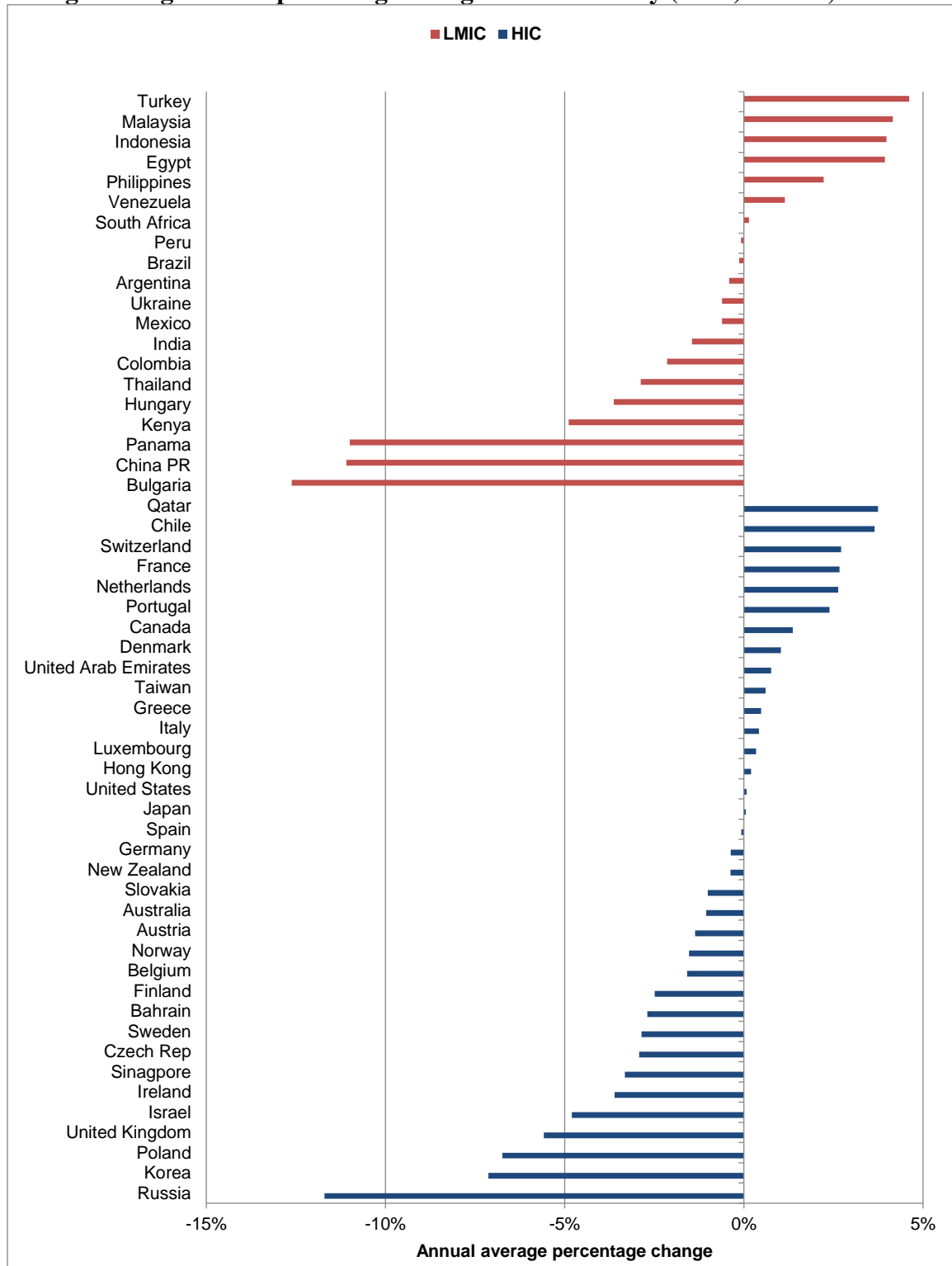

Supplement: S2 Fig — (PDF) [file pone.0208831.s003.pdf]
